# Integrated Analysis of Transcriptomics and Metabolomics Unveil the Novel Insight of One-Year-Old Precocious Mechanism in the Chinese Mitten Crab, *Eriocheir sinensis*

**DOI:** 10.3390/ijms241311171

**Published:** 2023-07-06

**Authors:** Lanmei Wang, Jiancao Gao, Xi Cao, Jinliang Du, Liping Cao, Zhijuan Nie, Gangchun Xu, Zaijie Dong

**Affiliations:** 1 Key Laboratory of Freshwater, Fisheries and Germplasm Resources Utilization, Freshwater Fisheries Research Centre of Chinese Academy of Fishery Sciences, Ministry of Agriculture and Rural Affairs, Wuxi 214081, China; wanglm@ffrc.cn (L.W.); gaojc@ffrc.cn (J.G.); dujl@ffrc.cn (J.D.); caolp@ffrc.cn (L.C.); niezj@ffrc.cn (Z.N.); 2 Wuxi Fisheries College, Nanjing Agricultural University, Wuxi 214081, China; 3 College of Fisheries and Life Science, Shanghai Ocean University, Shanghai 201306, China; cxcaoxi111111@163.com

**Keywords:** precocity, transcriptomics, metabolomics, amino acids, fatty acids, flavorful nucleotides, Chinese mitten crab (*Eriocheir sinensis*)

## Abstract

*Eriocheir sinensis* is traditionally a native high-value crab that is widely distributed in eastern Asia, and the precocity is considered the bottleneck problem affecting the development of the industry. The precocious *E. sinensis* is defined as a crab that reaches complete sexual maturation during the first year of its lifespan rather than as normally in the second year. However, the exact regulatory mechanisms underlying the precocity are still unclear to date. This study is the first to explore the mechanism of precocity with transcriptome-metabolome association analysis between the precocious and normal sexually mature *E. sinensis*. Our results indicated that the phenylalanine metabolism (map00360) and neuroactive ligand-receptor interaction (map04080) pathways play an important role in the precocity in the ovary of *E. sinensis*. In map00360, the predicted aromatic-L-amino-acid decarboxylase and 4-hydroxyphenylpyruvate dioxygenase isoform X1 genes and the phenethylamine, phenylethyl alcohol, trans-2-hydroxycinnamate, and L-tyrosine metabolites were all down-regulated in the ovary of the precocious *E. sinensis*. The map04080 was the common KEGG pathway in the ovary and hepatopancreas between the precocious and normal crab. In the ovary, the predicted growth hormone secretagogue receptor type 1 gene was up-regulated, and the L-glutamate metabolite was down-regulated in the precocious *E. sinensis*. In the hepatopancreas, the predicted forkhead box protein I2 gene and taurine metabolite were up-regulated and the the L-glutamate metabolite was down-regulated in the precocious crab. There was no common pathway in the testis. Numerous common pathways in the hepatopancreas between male precocious and normal crab were identified. The specific amino acids, fatty acids and flavorful nucleotide (inosine monophosphate (MP), cytidine MP, adenosine MP, uridine MP, and guanosine MP) contents in the hepatopancreas and gonads further confirmed the above omics results. Our results suggest that the phenylalanine metabolism may affect the ovarian development by changing the contents of the neurotransmitter and tyrosine. The neuroactive ligand–receptor interaction pathway may affect the growth by changing the expressions of related genes and affect the umami taste of the gonads and hepatopancreas through the differences of L-glutamate metabolite in the precocious *E. sinensis*. The results provided valuable and novel insights on the precocious mechanism and may have a significant impact on the development of the *E. sinensis* aquaculture industry.

## 1. Introduction

The Chinese mitten crab *Eriocheir sinensis* is traditionally a native high-value freshwater crab that is widely distributed in eastern Asia, particularly in China and Korea [1,2]. During the past few decades, there has been a significant increase in the aquaculture of this species in China [3,4] because of high market demand due to its taste and the advances in hatchery and grow out techniques. The aquaculture yields have reached approximately 808,274 tonnes in 2021 of China. Although the culture of *E. sinensis* has become a large industry, the precocity is considered the bottleneck problem affecting the development of the industry [5]. The precocious *E. sinensis* is defined as crabs that reach complete sexual maturation during the first year of their lifespan rather than normally in the second year [6]. The occurrence of precocious crabs commonly accounts for 15–30% of crabs when they are harvested in December [7]. Molting and growth are terminated in the precocious *E. sinensis*, which causes significant economic loss to farmers and leads to germplasm degradation [8].

Studies have shown that the precocity of *E. sinensis* is linked to many factors, including over-nutrition and external environmental factors, such as temperature and salinity, as well as internal factors, such as species and endocrine regulation [9,10]. On over-nutrition, Lu et al. (2010) [11] and Wu et al. (2011) [12] indicated that an unbalanced dietary lipid composition was a prominent factor for precocity. The restriction of feeding or fasting, which is used to control the precocious rate of the crab’s maturation in practice, usually results in small size and low market price [13]. Researchers suggested that nutrition is transferred from the hepatopancreas to the gonads during the process of the precocity in the Chinese mitten crab [14,15]. Xu et al. (2017) [8] speculated that the abnormal physiological activities in the hepatopancreas might result in the precocity of the juvenile *E. sinensis*. Increased fat storage in the hepatopancreas and a lack of phospholipid intake reduced protein utilization, which caused early molting, followed by precocity [16]. External environmental factors, such as temperature, light intensity and photoperiod are believed to play an important role in controlling the maturation of crustaceans [5]. The dim light was favored for the ovarian maturation of prawns, and bright light had a strong inhibitory effect on developed ovaries [17]. Internal factors, such as the disordered secretion of various hormones by the endocrine system, may lead to precocity [18,19]. However, the exact regulatory mechanisms underlying the precocity of the *E. sinensis* are still unclear.

The precocity of crabs is a complicated biological process that involves the interaction of multiple genes and metabolic pathways. Recently, integrated transcriptomic and metabolomic analysis reported on *Coilia nasus* [20], *Pinctada fucata martensii* [21], *Plectropomus leopardus* [22] and *E. sinensis* [23] discovered the biological rules with diverse phenotypes. The comprehensive analysis of metabolomics and transcriptomics in *E. sinensis* provided novel insights into the molecular and metabolic mechanisms in controlling growth during the molting cycle [23]. Herein, we conducted untargeted liquid chromatography-mass spectrometry (LC–MS) and RNA sequencing to investigate differences in metabolomic and transcriptomic profiles between precocious one-year-old and normal two-year-old sexually mature *E. sinensis* individuals. The findings of this study will provide valuable and novel insights on the precocious mechanism, which may have a significant impact on the development of the *E. sinensis* aquaculture industry.

## 2. Results

### 2.1. Overview of RNA-Sequencing and Assembly

**Figure 1:**
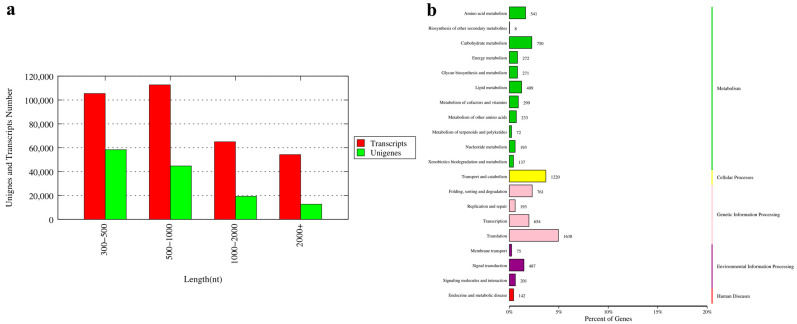
The length distribution of unigenes (**a**) and Kyoto Encyclopedia of Genes and Genomes (KEGG) annotation pathway of unigenes (**b**) for the transcriptome of *E. sinensis*. The vertical coordinate (left) is the name of KEGG secondary metabolic pathway, the vertical coordinate (right) is the name of KEGG primary metabolic pathway, and the horizontal coordinate is the number of genes annotated to this pathway and their proportion to the total number of genes annotated.

In this study, 36 cDNA libraries of three tissues (eyestalk E, hepatopancreas H, and gonads G) of the male (M) and female (F) one-year-old precocious (P) and two-year-old normal (N) sexually mature *E. sinensis* were constructed and sequenced, with three bio-replicates of each sample. The samples were named PFE, PFH, PFG, PME, PMH, PMG, NFE, NFH, NFG, NME, NMH and NMG, respectively. Each library was sequenced on the Nova seq6000 platform. A total of 248.78 Gb clean data were obtained, with more than 5.95 Gb of each sample. The Q30 ratio was larger than 90.8% for all samples (Supplementary Table S1). All the data are available at NCBI SRA database (PRJNA897950). The final de novo assembly set consisted of 337,221 transcripts and 135,007 unigenes. The N50 of the transcripts and unigenes were 1964 and 1290, respectively. There were 31,975 (23.68%) unigenes longer than 1000 bp (Figure 1a).

All clean reads of each sample were compared with the assembled reference genome, and the mapping rate ranged from 62.85% to 86.64%. All unigenes were compared with seven databases including Nr (NCBI non-redundant protein sequences), the UniProt-SwissProt (a manually annotated and reviewed protein sequence database), KEGG (Kyoto Encyclopedia of Genes and Genomes), KOG (euKaryotic Orthologous Groups), COG (Cluster of Orthologous Groups of protein), GO (Gene Ontology) and Pfam (Protein family) database for functional annotation. A total of 54,441 unigene annotation results were obtained. All the annotation results are provided in Supplementary Table S1. To identify the biological pathway in the Chinese mitten crab transcriptome, all contigs were mapped to the KEGG database. The top 20 pathways with the largest group of contigs are shown in Figure 1b. Among the top 20 pathways, more than half of the pathways focused on metabolism, followed by genetic information processing and environmental information processing.

### 2.2. Differential Expression of mRNAs

To investigate the differential expression levels of mRNAs between the precocious and normal mature crab, six comparison groups (PFE_vs_NFE, PFG_vs_NFG, PFH_vs_NFH, PME_vs_NME, PMG_vs_NMG, and PMH_vs_NMH) were applied to identify differentially expressed mRNAs (DE-mRNAs). Based on the criteria that Fold Change (FC) ≥ 2 and False Discovery Rate (FDR) < 0.5, we identified 915, 359, 348, 3776, 197 and 332 differentially expressed genes (DEGs) in PFE_vs_NFE, PFG_vs_NFG, PFH_vs_NFH, PME_vs_NME, PMG_vs_NMG and PMH_vs_NMH, respectively.

**Figure 2:**
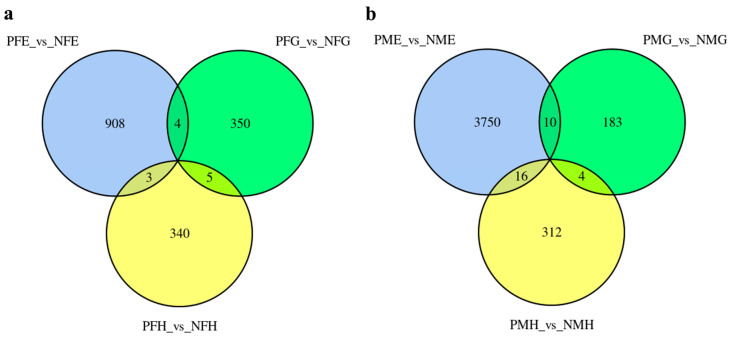
The differentially expressed genes (DEGs) number and Venn diagram of the overlap of the three PFE_vs_NFE, PFG_vs_NFG, and PFH_vs_NFH groups (**a**) and of the PME_vs_NME, PMG_vs_NMG, and PMH_vs_NMH groups (**b**).

Further analysis indicated that no unigene showed significantly different expression levels in all three PFE_vs_NFE, PFG_vs_NFG, and PFH_vs_NFH groups (Figure 2a) and PME_vs_NME, PMG_vs_NMG, and PMH_vs_NMH groups (Figure 2b). There were five and four of the same DEGs in the two PFG_vs_NFG, PFH_vs_NFH groups and PMG_vs_NMG, PMH_vs_NMH groups (Figure 2a,b), respectively, but no annotation information was found for the nine genes. All the results of DEGs annotation are listed in Supplementary Table S2.

### 2.3. Enrichment and Pathway Analysis of DEGs

**Figure 3:**
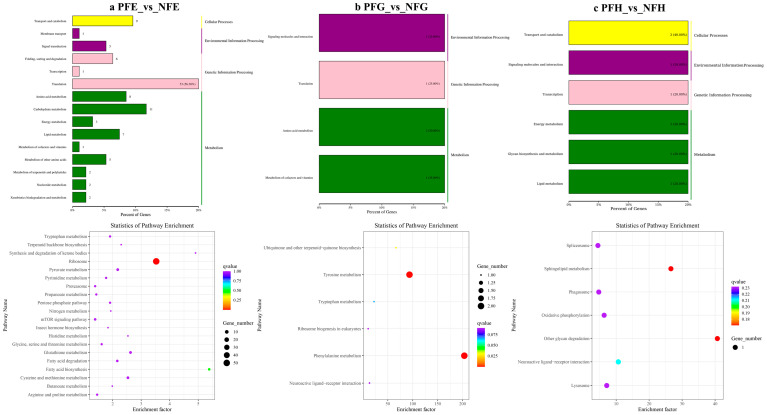
KEGG classification (**up**) and enrichment scatterplot (**down**) of differentially expressed genes (DEGs) in PFE_vs_NFE (**a**), PFG_vs_NFG (**b**), PFH_vs_NFH (**c**). The abscissa of the scatterplot is the enrichment factor, which represents the ratio of the number of target genes divided by the number of all the genes in each pathway. The larger the enrichment factor, the more significant the enrichment level of DEGs in this pathway. The color of the dot represents the q value, and the size of the dot represents the number of DEGs in the pathway. The same below.

**Figure 4:**
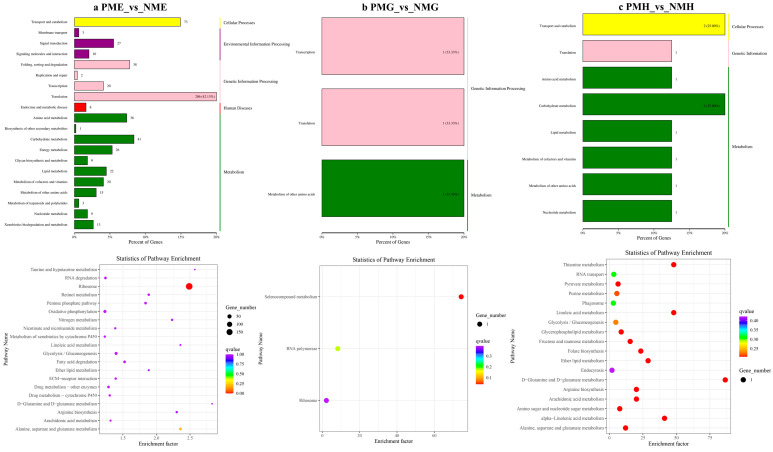
KEGG classification (**up**) and enrichment scatterplot (**down**) of DEGs in PME_vs_NME (**a**), PMG_vs_NMG (**b**) and PMH_vs_NMH (**c**).

In the further analysis of the GO term enrichment and KEGG pathway, all the DEGs were classified into different gene ontologies and pathways. The KEGG DEGs annotation results of the six groups (PFE_vs_NFE, PFG_vs_NFG, PFH_vs_NFH, PME_vs_NME, PMG_vs_NMG, and PMH_vs_NMH) are shown in Supplementary Table S3. Among the four biological processes, including cellular processes, environmental information processing, genetic information processing, and metabolism, the translation, the genetic information processing, had the highest number of DEGs in the PFE_vs_NFE (56.38%) (Figure 3a) and the PME_vs_NME (42.13%) group (Figure 4a), respectively, and ribosome was the main enrichment pathway (Figure 3a and Figure 4a). The KEGG DEGs of PFG_vs_NFG and PMG_vs_NMG were very few. Phenylalanine metabolism and tyrosine metabolism were the main enrichment pathways in PFG_NFG (Figure 3b), and selenocompound metabolism was the notable enrichment pathway in PMG_NMG (Figure 4b). In PFH_NFH, the main enrichment pathways included sphingolipid metabolism and other glycan degradation (Figure 3c). In PMH_NMH, most of the DEGs were also enriched in the metabolic pathways, including D-Glutamine and D-glutamate metabolism, thiamine metabolism, linoleic acid metabolism, ether lipid metabolism. and so on (Figure 4c).

### 2.4. Differential Metabolites Analysis

In this study, the metabolomics analysis was performed of 12 samples in *Eriocheir sinensis*, with six bio-replicates of each sample. The database mapping results of all metabolites, including numbering index, classification and pathway information, are shown in Supplementary Table S4. Six comparative analyses including PFS (serum) (hemolymph)_vs_NFS, PFH_vs_NFH, PFG_vs_NFG, PMS_vs_NMS, PMH_vs_NMH and PMG_vs_NMG were performed with the positive ion mode (POS) and the negative ion mode (NEG). All the data in this study has been submitted to MetaboLights (MTBLS3686). 

The POS results of the differential metabolite screening of each comparative group are listed in Supplementary Table S5. We identified 70, 80, 43, 100, 61 and 84 significant differential metabolites (SDMs) in PFS_vs_NFS, PFG_vs_NFG, PFH_vs_NFH, PMS_vs_NMS, PMG_vs_NMG and PMH_vs_NMH, respectively.

**Figure 5:**
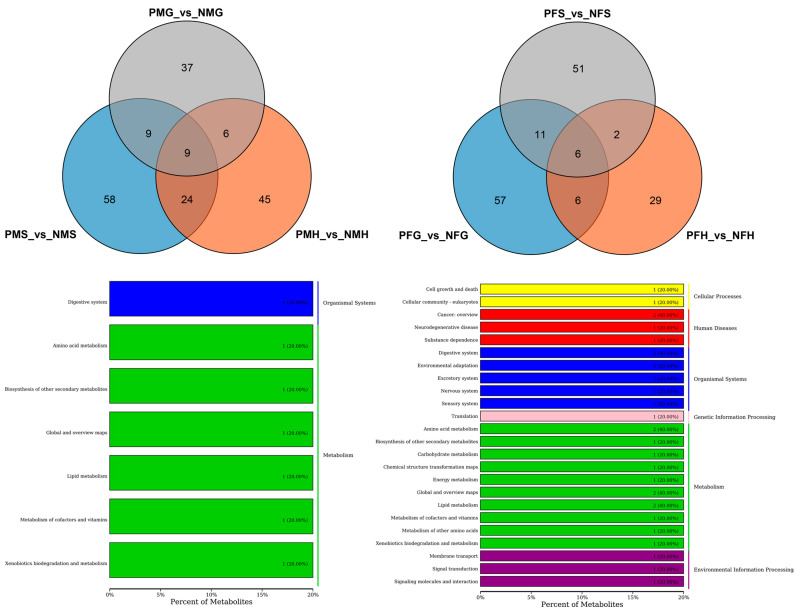
Differential metabolites number and Venn diagram of overlap (**Up**) of the different groups with positive ion mode (POS). And the KEGG classification (**Down**) of the same significant differential metabolites (SDMs) between PMG_vs_NMG and PMH_vs_NMH (**left**), and PFG_vs_NFG and PFH_vs_NFH (**right**) with POS mode.

Further analysis found that there were nine shared SDMs in all the three PMS_vs_NMS, PMG_vs_NMG and PMH_vs_NMH groups and six shared SDMs in all the PFS_vs_NFS, PFG_vs_NFG and PFH_vs_NFH groups (Figure 5). The nine shared SDMs were L-urobilin, 4-methylbenzaldehyde, safrole, fragransin B2, dehydroepiandrosterone sulfate, delta-tetradecalactone, 2-phenylethanol, norvaline and 2,6-toluenediamine, which are involved in the degradation of aromatic compounds, xylene degradation, steroid hormone biosynthesis, metabolic pathways, phenylalanine metabolism, bile secretion, microbial metabolism in diverse environments, and porphyrin and chlorophyll metabolism KEGG pathways. The six shared SDMs were fragransin B2, dehydroepiandrosterone sulfate, glycerol tripropanoate, proline betaine, 2-phenylethanol and 4-methylbenzaldehyde, which are involved in the steroid hormone biosynthesis, bile secretion, phenylalanine metabolism, microbial metabolism in diverse environments, xylene degradation, metabolic pathways, and degradation of aromatic compounds KEGG pathways. Of particular interest are shared SDMs between PFG_vs_NFG and PFH_vs_NFH, and between PMG_vs_NMG and PMH_vs_NMH, and twelve and fifteen shared SDMs were found, respectively (Figure 5). The KEGG pathways of the same SDMs between PMG_vs_NMG and PMH_vs_NMH focused on the phenylpropanoid biosynthesis, bile secretion, steroid hormone biosynthesis, metabolic pathways, phenylalanine metabolism, xylene degradation, porphyrin and chlorophyll metabolism, degradation of aromatic compounds, and microbial metabolism in diverse environments, most of which were shared to that in the three PMS_vs_NMS, PMG_vs_NMG and PMH_vs_NMH groups. The pathways between PFG_vs_NFG and PFH_vs_NFH were mainly in microbial metabolism in diverse environments and metabolic pathways including amino acid metabolism, lipid metabolism and so on (Figure 5).

The NEG results of differential metabolite screening of each comparative group are listed in Supplementary Table S6. The NEG results had fewer SDMs of each comparative group than the POS results. We identified 18, 45, 13, 51, 18 and 28 SDMs in PFS_vs_NFS, PFG_vs_NFG, PFH_vs_NFH, PMS_vs_NMS, PMG_vs_NMG, and PMH_vs_NMH, respectively.

**Figure 6:**
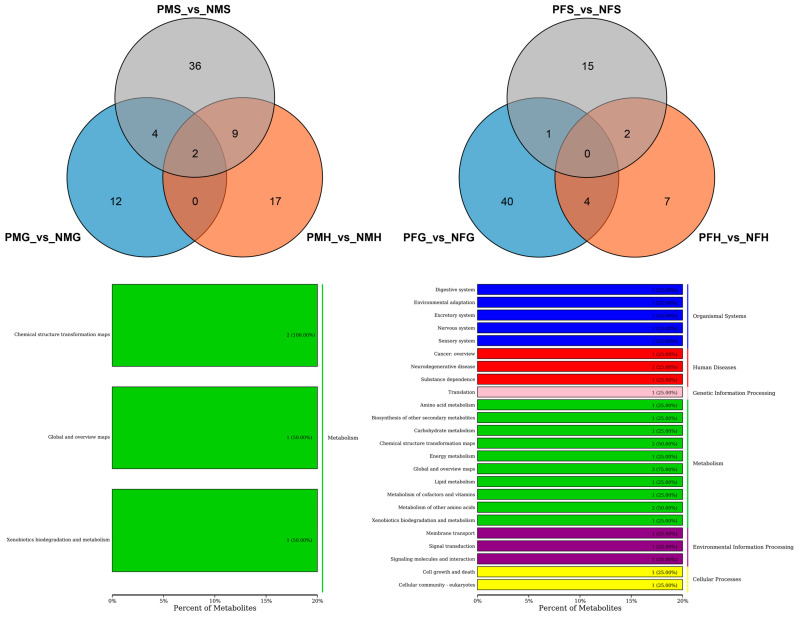
Number of differential metabolites and Venn diagram of overlap of the different groups with negative ion mode (NEG) (**Up**). KEGG classification (**Down**) of the SDMs between PMG_vs_NMG and PMH_vs_NMH (**left**), and PFG_vs_NFG and PFH_vs_NFH (**right**) with NEG mode.

Further analysis indicated that there were just two shared SDMs in all the three PMS_vs_NMS, PMG_vs_NMG and PMH_vs_NMH groups, and no metabolite showed significantly different in all the three PFS_vs_NFS, PFG_vs_NFG and PFH_vs_NFH groups (Figure 6). The two shared SDMs were gallic acid and marmesin, which are involved in the aminobenzoate degradation, biosynthesis of phenylpropanoids, and microbial metabolism in diverse environments of KEGG pathways. The two shared SDMs between PMG_vs_NMG and PMH_vs_NMH were the same as those in the three PMS_vs_NMS, PMG_vs_NMG and PMH_vs_NMH groups. The KEGG pathways of the four shared SDMs between PFG_vs_NFG and PFH_vs_NFH mainly focused on the metabolic pathways, microbial metabolism in diverse environments, biosynthesis of secondary metabolites, D-glutamine, and D-glutamate metabolism, etc. (Figure 6).

### 2.5. Enrichment and Pathway Analysis of SDMs

**Figure 7:**
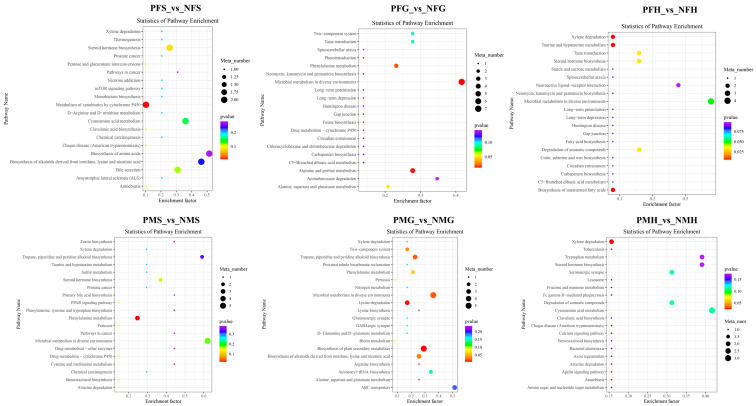
KEGG enrichment scatterplot of the SDMs in PFS_vs_NFS, PFG_vs_NFG, PFH_vs_NFH, PMS_vs_NMS, PMG_vs_NMG, and PMH_vs_NMH with POS mode.

In POS analysis mode, the KEGG SDMs annotation results of the six groups are shown in Supplementary Table S7. The metabolic KEGG pathway types had the highest number of SDMs in each comparative group. Further pathway enrichment analysis of the SDMs wasperformed. As shown in Figure 7, the metabolism of xenobiotics by cytochrome P450 and steroid hormone biosynthesis were the main enrichment pathways in PFS_NFS. In PFG_NFG, the microbial metabolism in diverse environments, arginine and proline metabolism, and phenylalanine metabolism were the main enrichment pathways. In PFH_NFH, most of the SDMs were enriched in the xylene degradation, the biosynthesis of unsaturated fatty acids, taurine and the hypotaurine metabolism pathways. Phenylalanine metabolism was the main enrichment pathway in PMS_NMS. In PMG_NMG, the enrichment pathways included biosynthesis of plant secondary metabolites, lysine degradation, microbial metabolism in diverse environments, tropane, piperidine, and pyridine alkaloid biosynthesis, etc. Xylene degradation and cyanoamino acid metabolism were the main enrichment pathways in PMH_NMH.

**Figure 8:**
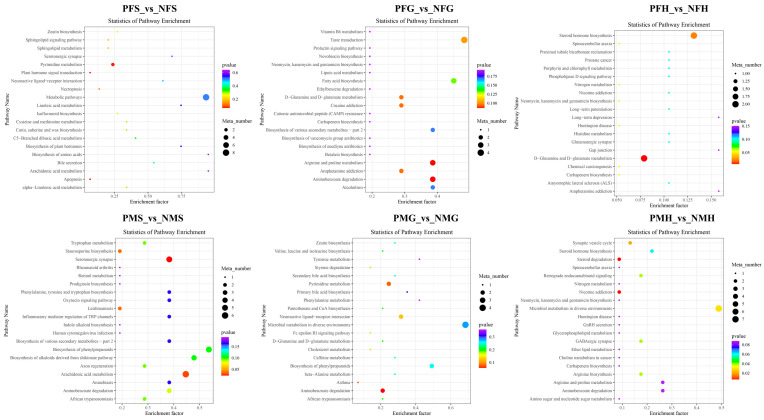
KEGG enrichment scatterplot of the SDMs in PFS_vs_NFS, PFG_vs_NFG, PFH_vs_NFH, PMS_vs_NMS, PMG_vs_NMG, and PMH_vs_NMH with NEG mode.

In the NEG analysis mode, the KEGG SDMs annotation results of the six groups are shown in Supplementary Table S8. The pathway bubble plots of the SDMs in the six comparative groups are shown in Figure 8. In PFS_NFS, the apoptosis, plant hormone signal transduction, and pyrimidine metabolism were the main enrichment pathways. In PFG_NFG, the enrichment pathways included aminobenzoate degradation, arginine and proline metabolism, amphetamine addiction, D-Glutamine and D-glutamate metabolism, cocaine addiction, taste transduction, etc. The D-Glutamine and D-glutamate metabolism and steroid hormone biosynthesis were the main enrichment pathways in PFH_NFH. In PMS_NMS, most of the SDMs were enriched in the serotonergic synapse and arachidonic acid metabolism. Amino benzoate degradation, asthma, and pyrimidine metabolism were the main enrichment pathways in PMG_NMG, and nicotine addiction and steroid degradation were the main enrichment pathways in PMH_NMH.

### 2.6. Transcriptomics-Metabolomics Co-Regulated Network Analysis

In this study, the spearman correlation coefficient was calculated according to the characteristic vector value of the metabolite module and the expression amount of differential genes in the PFH_vs_NFH, PFG_vs_NFG, PMH_vs_NMH, and PMG_vs_NMG groups. The spearman correlation heatmaps of each comparison group are shown in Supplementary Figure S1a, respectively. Next, the DEGs-SDMs network regulation relationship was performed. The relationship of network regulation is very complicated (Supplementary Figure S1b).

We further screened the common KEGG pathways in transcriptome and metabonomics analysis and marked the differential genes and metabolites on the KEGG pathway maps. The detailed results are shown in Supplementary Table S9; there was no common pathway in the PMG_vs_NMG group with POS and NEG analysis. In POS mode, the common pathway of the DEGs and differential metabolites was phenylalanine metabolism (map00360), tryptophan metabolism (map00380) and neuroactive ligand-receptor interaction (map04080) in PFG_vs_NFG. In PFH_vs_NFH, the map04080 was the only common KEGG pathway. In PMH_vs_NMH, the fructose and mannose metabolism (map00051), arginine biosynthesis (map00220), purine metabolism (map00230), amino sugar and nucleotide sugar metabolism (map00520), glycerophospho lipid metabolism (map00564), and arachidonic acid metabolism (map04080) were the common pathways. In NEG mode, the common pathway of the DEGs and differential metabolites was ubiquinone and other terpenoid-quinone biosynthesis (map00130), tyrosine metabolism (map00350), map00360, and map04080 in PFG_vs_NFG. In PFH_vs_NFH, the map04080 was also the only common KEGG pathway. In PMH_vs_NMH, the map00220, alanine, aspartate and glutamate metabolism (map00250), D-glutamine and D-glutamate metabolism (map00471), map00520, map00564, ether lipid metabolism (map00565), linoleic acid metabolism (map00591), and α-linolenic acid metabolism (map00592) were the common pathways

**Figure 9:**
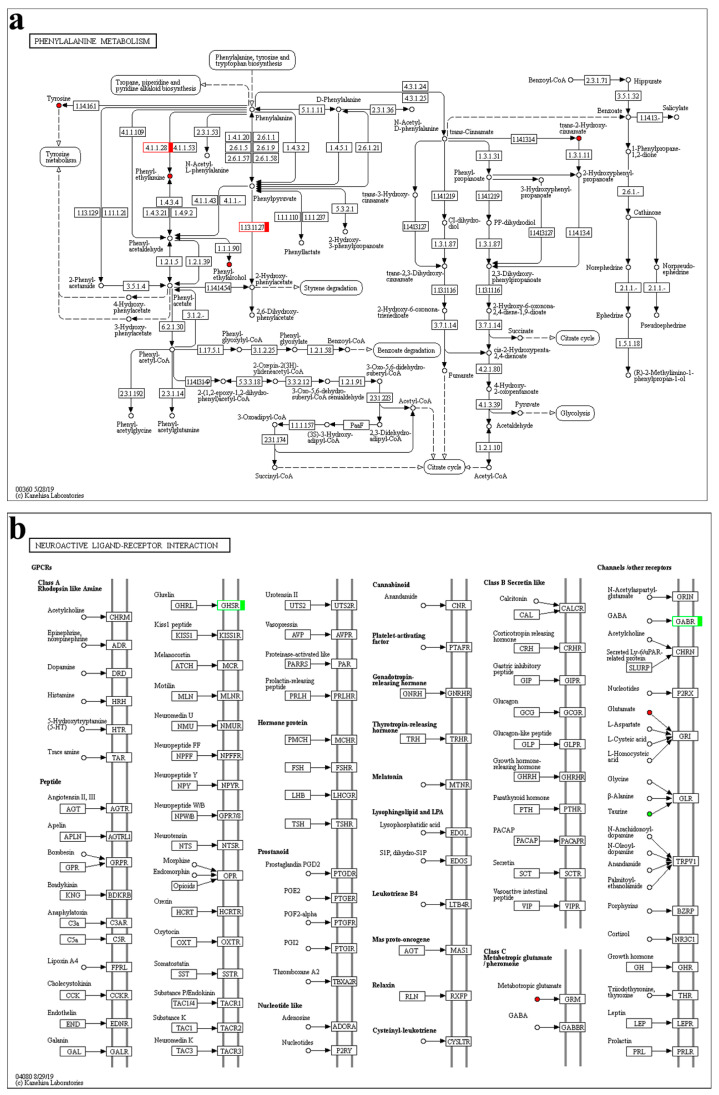
The phenylalanine metabolism (**a**) and neuroactive ligand-receptor interaction (**b**) KEGG pathway. Red indicates up-regulation; green indicates down-regulation.

Overall, the phenylalanine metabolism (Figure 9a) and neuroactive ligand-receptor interaction pathways (Figure 9b) were crucial in PFG_vs_NFG, combining the transcriptome and metabolome results. In map00360, the predicted aromatic-L-amino-acid decarboxylase (TRINITY_DN4191_c7_g1) and 4-hydroxyphenylpyruvate dioxygenase isoform X1 (TRINITY_DN60264_c0_g1) genes and the phenethylamine, phenylethyl alcohol, trans-2-hydroxycinnamate and L-tyrosine metabolites were all up-regulated in PFG_vs_NFG. In map04080, the predicted growth hormone secretagogue receptor type 1 (TRINITY_DN42303_c0_g1) gene was down-regulated and the L-glutamate metabolite was up-regulated in PFG_vs_NFG. The map04080 was also the only common KEGG pathway in PFH_vs_NFH, and the predicted forkhead box protein I2 (TRINITY_DN9364_c0_g2) gene and taurine metabolite were down-regulated and the the L-glutamate metabolite was up-regulated.

There was no common pathway in PMG_vs_NMG. In PMH_vs_NMH, the pathways were complex, which mainly focused on the amino acid, lipid, and energy metabolism, such as arginine biosynthesis, amino sugar and nucleotide sugar metabolism, and glycerophospholipid metabolism.

### 2.7. Specific Amino Acids, Fatty Acids and Flavorful Nucleotides Content Analysis

To further confirm and understand the above results, we randomly chose six crabs in the male and female one-year precocious and two-year normally mature groups to obtain the hepatopancreas and gonads. The free amino acids, fatty acids, and flavorful nucleotide contents were detected. As shown in Table 1, the phenylalanine (Phe) content was higher in PFG than in NFG. On the contrary, the tyrosine (Tyr) content was lower in PFG than in NFG. In the PMH_vs_NMH group, the cysteine (Cys-s) contents in PFH and PMH were higher than in NFH and NMH, respectively. The arginine (Arg), glutamic acid (Glu), and glutamine (Gln) contents in PMH were all higher than that in NMH. The gln content in PFH was also higher than in NFH. The differences of these above amino acids all corresponded to the amino acid-related enrichment KEGG pathways of DEGs in transcriptome and SDMs in metabolome.

For fatty acid, the linoleic acid (C18:2n6c) contents in NFG, NMH and NMG were higher than that in PFG, PMH and PMG, respectively. The C18:3n3 trioctadeca content in PMH was higher than that in NMH (Table 2). The n-6 polyunsaturated fatty acid (PUFAs) in NFH, NFG, NMH, and NMG was all higher than in PFH, PFG, PMH and PMG, respectively. On the contrary, the n-3 PUFAs and n-3/n-6 PUFAs in NFH, NFG, NMH, and NMG were all lower than in PFH, PFG, PMH and PMG, respectively (Table 2). Significantly, the arachidonic acid (C20:4n6) contents in NFH and NFG were also higher than that in PFH and PFG, respectively (Table 2).

As shown in Table 3, the inosine monophosphate (IMP) contents in NFG and NMG were higher than those in PFG and PMG, respectively. However, the cytidine monophosphate (CMP), adenosine monophosphate (AMP), uridine monophosphate (UMP), and guanosine monophosphate (GMP) in NFG were all lower than in PFG. The flavorful nucleotide contents of the gonads in the female crab were higher than those in the male crab, and the NMG crab had more UMP content than the PMG crab. The flavorful nucleotide contents in the hepatopancreas of the crab were relatively low, except for the GMP.

## 3. Discussion

Sexual precocity is a serious bottleneck problem in the aquaculture of *E. sinensis*, and the genetic mechanism of precocity is not currently well understood. Precocity is also a complex biological process involving interaction of multiple genes and metabolic pathways to induce early gonad development [24]. To our knowledge, the present study is the first to explore the mechanism of precocity with genes-metabolites association analysis in *E. sinensis*. In this study, we found that the phenylalanine metabolism and neuroactive ligand-receptor interaction pathways played an important role in the precocity in the ovary of *E. sinensis*. In the phenylalanine metabolism pathway, Phe is converted to Tyr through a hydroxylation reaction, which provides a precursor for the synthesis of hormones and neurotransmitters involving dopamine, norepinephrine, epinephrine, and thyroid hormone [25]. When phenylalanine hydroxylase defects occur, Phe generates phenylpyruvic acid, phenylacetic acid, and N-acetyl-L-phenylalanine metabolisms, and an abnormal accumulation of the byproducts of phenylalanine metabolism can be seen in the urine and plasma of phenylketonuria patients, which is the most common amino acid metabolism defect [26,27,28]. Phe deficiency will affect Tyr synthesis and cause a decrease in thyroxine levels, affecting the normal metabolic activities of animals [29]. In the comparative analysis of PFG_vs_NFG, the expression level of aromatic-L-amino-acid decarboxylase and 4-hydroxyphenylpyruvate dioxygenase isoform X1 gene and the phenethylamine, phenylethyl alcohol, trans-2-hydroxycinnamate, and L-tyrosine metabolites were all up-regulated in the phenylalanine metabolism pathway. The results implied that the phenylalanine metabolism may be disturbed or inhibited in the ovary of precocious crabs.

Aromatic L-amino acid decarboxylase is an enzyme that is essential for the conversion of 5-hydroxytryptophan to 5-hydroxytryptamine and of L-dopa to dopamine. It is also involved in the synthesis of trace amines such as tyramine from tyrosine, 2-phenylethylamine from phenylalanine and tryptamine from tryptophan [30]. The 4-hydroxyphenylpyruvate dioxygenase catalyzes the second step in the pathway for the catabolism of tyrosine, which yields acetoacetate and fumarate from L-tyrosine [31]. While these ketogenic and glucogenic products make a direct energetic contribution, they are required to modulate blood tyrosine levels in animals [32]. In humans, deficiencies of specific enzymes of the tyrosine catabolism pathway give rise to a number of severe metabolic disorders [33]. In this study, the down-regulated results of aromatic-L-amino-acid decarboxylase and 4-hydroxyphenylpyruvate dioxygenase isoform X1 gene expression and the phenethylamine, phenylethyl alcohol, trans-2-hydroxycinnamate, and L-tyrosine metabolites in the ovary of the precocious *E. sinensis*, suggested that the phenylalanine metabolism may affect ovarian development by changing the contents of the neurotransmitter and tyrosine. Correspondingly, the amino acid of Phe content was higher and Tyr content was lower in the ovary of precocious one-year-old crab than normal two-year-old sexually mature *E. sinensis.*

The neuroactive ligand–receptor interaction signaling pathway was directly related to neuro function [34]. Neuroactive ligands influence neuronal function by binding to intracellular receptors, which has the capability of binding transcription factors and regulating gene expressions [35]. In this study, the neuroactive ligand-receptor interaction pathway was all the common KEGG pathway in PFG_vs_NFG and PFH_vs_NFH with transcriptome and metabonomics association analysis. In the ovary, the growth hormone secretagogue receptor type 1 (GHS-R1) gene was up-regulated, and the L-glutamate metabolite was down-regulated in the precocious *E. sinensis.* The activation of the GHS-R1a by ghrelin stimulates feeding and growth hormone release, among other functions [36]. Naturally occurring mutations of the GHS-R1a in humans have been connected to growth disorders, metabolic syndrome, and obesity [37,38]. Significantly, ghrelin’s role is in the regulation of glucose homeostasis, and GHS-R1a is co-expressed with dopamine receptors in the brain [39,40]. The L-glutamate, inosinate (IMP) and guanosinate (GMP) and so on can contribute to the umami taste [41], and there is a synergistic effect between these umami compounds which can enhance the umami taste [42]. Correspondingly, the flavorful nucleotides of the IMP contents were lower in the gonads of precocious crabs than in normal sexually mature *E. sinensis.*

In the hepatopancreas, the forkhead box protein I2 gene and taurine metabolite was up-regulated, and the L-glutamate metabolite was down-regulated in the precocious *E. sinensis.* The forkhead box is a family of transcription factors that are involved in different cellular processes such as oxidative stress resistance, cell proliferation, differentiation, metabolism, and apoptosis [43,44,45,46,47]. Taurine, a special amino acid with a sulfur-content non-protein structure, is mainly distributed in some excitable tissues, such as the liver, central nervous system, heart, and skeletal muscle [48]. More importantly, taurine can protect the liver, improve the gallbladder, and regulate blood pressure as an antitumor, anti-oxidant, and anti-arrhythmia compound [49,50,51,52]. Corresponding to the result of down-regulated of L-glutamate, the IMP content was lower in the hepatopancreas of female precocious crab. The above results implied that the neuroactive ligand–receptor interaction pathway may affect the growth by changing the expressions of related genes and affect the umami taste of the gonads and hepatopancreas through the differences of L-glutamate metabolite in precocious *E. sinensis.*

Transcriptome-metabolome association analysis showed no common pathway in the testes betwwen precocious and normal sexually mature *E. sinensis*. The common pathways were complex in the comparative analysis of PFG_vs_NFG, which mainly focused on the amino acid, lipid, and energy metabolism, such as arginine biosynthesis, amino sugar and nucleotide sugar metabolism, and glycerophospholipid metabolism. Correspondingly, the specific amino acid of Arg and lipid acid of C18:3n3 trioctadeca contents were different in the hepatopancreas of male precocious and normal sexually mature *E. sinensis.* Chen et al. (2019) [24] reported the important function of the hepatopancreas and eyestalk in regulating the ovary development of *E. sinensis*. It is accepted that the hepatopancreas is an essential organ for energy storage and metabolism in the process of growth and development of crustaceans [53,54]. The synthesis and metabolism of certain steroid hormones required by crustaceans also occur in the hepatopancreas during vitellogenesis [55]. In this study, just the ribosome was the main enrichment pathway of DEGs in the eyestalk for precocious and normal sexually mature crabs. The metabolism of xenobiotics by cytochrome P450, steroid hormone biosynthesis, apoptosis, plant hormone signal transduction, and pyrimidine metabolism were the main enrichment pathways of SDMs in PFS_NFS. In PMS_NMS, most of the SDMs are enriched in the phenylalanine metabolism, serotonergic synapse, and arachidonic acid metabolism. It can be seen that hormones secreted by the endocrine system play an important role in the process of precocity [18,19]. Furthermore, functional experiments need to be conducted to make more discoveries.

## 4. Materials and Methods

### 4.1. Ethics Statement

All animal experiments in this study were approved by the Bioethical Committee of Freshwater Fisheries Research Center (FFRC) of Chinese Academy of Fishery Sciences (CAFS) (BC 2013863, 9/2013), guidelines for the Care and Use of Experimental Animals of China.

### 4.2. Animal Sampling

**Figure 10:**
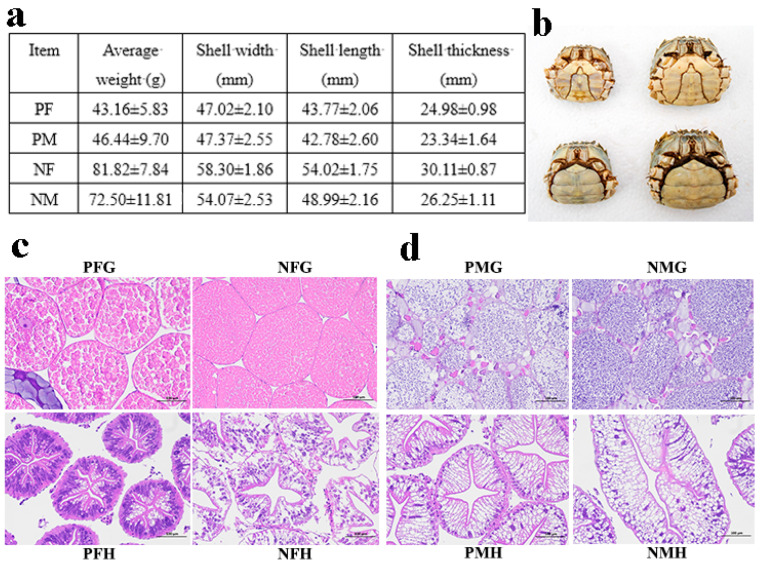
The phenotypic characteristics and histological observation of gonads and hepatopancreas of precocious and normal sexually mature *E. sinensis*. (**a**): the phenotypic characteristics of precocious female (PF), precocious male (PM), normal sexually mature female (NF) and normal mature male (NM) crabs. (**b**): the photograph of PF, PM, NF, and NM crabs. (**c**): PFG: gonads of precocious female *E. sinensis*; PFH: hepatopancreas of precocious female crab; NFG: gonads of normally mature female crab; NFH: hepatopancreas of normally mature female crab. (**d**): PMG: gonads of precocious male crab; PMH: hepatopancreas of precocious male crab; NMG: gonads of normally mature male crab; NMH: hepatopancreas of normally mature male crab. Scale bar: 100 µm.

In this study, the precocious one-year-old and normal two-year-old sexually mature *E. sinensis* individuals were collected from the Yangzhong Pilot Research Station (Zhenjiang, China) of FFRC, CAFS and were anesthetized on ice before sampling. We randomly selected 10 precocious female (PF), precocious male (PM), normal sexually mature female (NF) and normal sexually mature male (NM) crabs, respectively, to measure the phenotypic characters (Figure 10a,b). Then we further confirmed the gonadal and hepatopancreatic development stages by histological observation (Figure 10c,d). In this study, the eyestalk (E), hepatopancreas (H), and gonad (G) tissues of PF, PM, NF, and NM crabs were collected for transcriptome analysis, and the serum (S) (hemolymph), hepatopancreas, and gonad tissues of PF, PM, NF, and NM crabs were selected for metabolomics analysis. The hemolymph was let stand for 2 h at 4 °C and centrifuged at 4000 rpm for 10 min to obtain the serum. The samples were named as PFE, PFH, PFG, PME, PMH, PMG, NFE, NFH, NFG, NME, NMH, NMG, PFS, PMS, NFS, and NMS, respectively. All samples were immediately frozen in liquid nitrogen and then stored at −80 °C before used.

In addition, the hepatopancreas and gonad tissues of the crabs were fixed in PFA (phosphate buffered saline (PBS): formaldehyde = 9:1). Tissue blocks were dehydrated, cleared, embedded in paraffin, and sectioned for histological observation.

### 4.3. Transcriptome Analysis

The main analysis process included an RNA test, library construction, library quality control, sequencing, and bioinformatics analysis. 

The RNA for the eyestalk, hepatopancreas, and gonad tissues was extracted using the TRIZOL (Invitrogen, Carlsbad, CA, USA) method. RNA purity (OD260/280) was assessed using the Nanodrop-2000 spectrophotometer (Thermo Fisher Scientific, BRIMS, Cambridge, MA, USA). RNA integrity was assessed using the RNA Nano 6000 Assay Kit of the Agilent Bioanalyzer 2100 system (Agilent Technologies, Palo Alto, CA, USA). Next, 36 cDNA libraries of PFE, PFH, PFG, PME, PMH, PMG, NFE, NFH, NFG, NME, NMH, and NMG were constructed. Briefly, mRNA was purified from the total RNA using poly-T oligo-attached magnetic beads. The mRNA was randomly fragmented by a fragmentation buffer. The first strand of cDNA was synthesized using random hexamer primer; then second-strand cDNA synthesis was performed using DNA Polymerase I and RNase H. The purified double-stranded cDNA was then end-repaired, an A-tail added, and a sequencing adaptor connected. To select cDNA fragments in length, the library fragments were purified with AMPure XP system (Beckman Coulter, Beverly, MA, USA). Finally, the cDNA library was enriched by PCR.

The library quality was assessed on the Agilent Bioanalyzer 2100 system. The effective concentration of the library was accurately quantified by qPCR (the concentration > 2 nM) to complete the library inspection. Then the library preparations were sequenced on an Illumina Nova seq6000 platform for 150 bp paired-end sequencing.

The quality check of raw sequence reads was performed via FastQC v0.11.2 (http://www.bioinformatics.babraham.ac.uk/projects/fastqc/, accessed on 13 march 2022) [56]. Transcriptome assembly was accomplished using Trinity [57]. Gene function was annotated based on the following databases: Nr, the UniProt-SwissProt, KEGG, KOG, COG, GO, and Pfam database. Gene expression levels were estimated via RSEM [58] for each sample. The differential expression analysis of two samples was performed using the DEGseq (2010) R package.

GO enrichment analysis of the DEGs was implemented by the GOseq R packages- based Wallenius non-central hyper-geometric distribution [59], which can adjust for gene- length bias in DEGs. KEGG is a database resource for understanding high-level functions and utilities of the biological system. We used KOBAS 3.0 [60] software to test the statistical enrichment of differential expression genes in KEGG pathways.

### 4.4. Metabolome Analysis

In this study, the metabolomics analysis based on LC-MS/MS was performed on 12 samples in *E. sinensis*, with six bio-replicates of each sample. Six comparative analyses including PFS_vs_NFS, PFH_vs_NFH, PFG_vs_NFG, PMS_vs_NMS, PMH_vs_NMH, and PMG_vs_NMG were performed.

Metabolites Extraction: 100 μL of the sample was transferred to an EP tube. After the addition of 400 μL of extract solution (acetonitrile: methanol = 1:1, containing isotopically labeled internal standard mixture), the samples were vortexed for 30 s, sonicated for 10 min in an ice-water bath, and incubated for 1 h at −40 °C to precipitate proteins. Then 25 mg of sample was weighted to an EP tube after grinding with liquid nitrogen, and 500 μL extract solution (methanol:water = 3:1, with isotopically labelled internal standard mixture) was added. Then the samples were homogenized at 35 Hz for 4 min and sonicated for 5 min in an ice-water bath. The homogenization and sonication cycle was repeated 3 times. Then the samples were incubated for 1 h at −40 °C and centrifuged at 12,000 rpm (RCF = 13,800(× *g*), R = 8.6 cm) for 15 min at 4 °C. The resulting supernatant was transferred to a fresh glass vial for analysis. The quality control (QC) sample was prepared by mixing an equal aliquot of the supernatants from all the samples.

LC-MS/MS analysis: LC-MS/MS analysis was performed using an UHPLC system Q Exactive Orbitrap (Thermo Fisher Scientific, USA) with a UPLC BEH Amide column (2.1 mm × 100 mm, 1.7 μm) coupled to Orbitrap Exploris 120 mass spectrometer (Orbitrap MS, Thermo). The mobile phase consisted of 25 mmol/L ammonium acetate and 25 mmol/L acetic acid in water (A) and acetonitrile (B). The auto-sampler temperature was 4 °C, and the injection volume was 2 μL. The Orbitrap Exploris 120 mass spectrometer was used for its ability to acquire MS/MS spectra on information-dependent acquisition (IDA) mode in the control of the acquisition software (Orbitrap Tribrid MS 3.5SP1, Thermo). In this mode, the acquisition software continuously evaluates the full-scan MS spectrum. The ESI source conditions were set as follows: sheath gas flow rate as 50 Arb, Aux gas flow rate as 15 Arb, capillary temperature 320 °C, full MS resolution as 60,000, MS/MS resolution as 15,000, collision energy as 10/30/60 in NCE mode, spray Voltage as 3.8 kV (positive) or −3.4 kV (negative), respectively.

Data preprocessing and annotation: After the quality control of the raw data, the MS2 database was applied in metabolite annotation. The cutoff for annotation was set at 0.3. The ROPLS package of R language was utilized for multivariate statistical analyses. Autoscaling, mean-centering, and scaled to unit variance (UV) were performed before principal component analysis (PCA), Partial Least Squares-Discriminant Analysis (PLS-DA) and Orthogonal Projections to Latent Structures Discriminant Analysis (OPLS-DA) [61] to do automatic modeling analysis. Then, SDMs were identified by Variable Importance in the Projection, VIP > 1 and *p* < 0.05 [62] and analyzed with KOBAS and Fisher’s exact tests to explore the enriched KEGG pathways [63].

### 4.5. Transcriptomics-Metabolomics Co-Regulated Network Analysis

Spearman’s correlation coefficient for ranked data is a way to study the relationship between two variables based on ranked data. In this study, the spearman correlation coefficient was calculated according to the characteristic vector value of the metabolite module and the expression amount of differential genes in the PFH_vs_NFH, PFG_vs_NFG, PMH_vs_NMH, and PMG_vs_NMG groups. The interaction between differential genes and differential metabolites could be found through spearman correlation analysis to further explore the mechanism of strongly correlated genes and metabolites. First, we screened the top 50 DEGs with the lowest FDR values in the transcriptome and the top 50 differential metabolites with the largest VIP values in metabonomics. Then the Psych package of R project was used to calculate the spearman correlation between the top 50 DEGs and the top 50 differential metabolites [64], and it was visualized using a pheatmap package.

Based on the results of the transcriptome and metabolome KEGG pathways, the self-developed Perl program was used to screen the common pathways, and the differential genes and differential metabolites were labeled on the KEGG pathway map: up-regulation is shown in red, down-regulation is shown in green, and both up-regulation and down-regulation are shown in blue.

Gene-metabolic network regulation refers to the regulation map of the interaction between SDMs and DEGs. We can obtain the most important core SDMs and DEGs to further explore how the change of DEGs affects metabolites and leads to the change of the corresponding phenotype [65]. We first screened the top 200 DEGs with the lowest FDR values or the largest FC values in the transcriptome. Then the Psych package of R project (V3.6.2) was used to calculate the spearman correlation between the top 200 DEGs and the top 50 differential metabolites. The results with a correlation greater than 0.9 and correlation *p* value less than 0.01 were further screened and used to draw the network regulatory map. Cytoscape software (V3.7.2) was used to visualize the correlation results to obtain the network regulatory map; the rectangle represents DEGs, the ellipse represents SDMs, the blue line represents negative correlation, and the red line represents positive correlation.

### 4.6. Specific Amino Acids, Fatty Acids and Flavorful Nucleotides Content Analysis

The amino acid content was analyzed by Agilent liquid chromatography (Ag 1100). The fatty acid content was analyzed by Shimadzu GC-2030 gas chromatograph. The nucleotides content was analyzed by Waters2695 High Performance Liquid Chromatography (HPLC), and the column was Diamonsil C18 4.6 × 250 mm.

## 5. Conclusions

Sexual precocity is a serious bottleneck problem in the aquaculture of *E. sinensis*, and the genetic mechanism of precocity is not currently well understood. This study is the first to explore the mechanism of precocity with transcriptome-metabolome association analysis. We found that the phenylalanine metabolism and neuroactive ligand-receptor interaction pathways played an important role in the precocity in the ovary of *E. sinensis*. The phenylalanine metabolism may affect ovarian development by changing the contents of the neurotransmitter and tyrosine. The neuroactive ligand–receptor interaction pathway may affect growth by changing the expressions of related genes, and affect the umami taste of the gonads and hepatopancreas through the differences of L-glutamate metabolite in precocious *E. sinensis.* There was no common pathway in the testis, and the pathways were complex in the hepatopancreas between male precocious and normal sexually mature crabs. The specific amino acids, fatty acids, and flavorful nucleotide contents corresponded to the above omics results. Our results provided valuable and novel insights on the precocious mechanism, which may have a significant impact on the development of the *E. sinensis* aquaculture industry.

## Figures and Tables

**Table 1:** The free amino acid contents of the hepatopancreas and gonads in the precocious and normally mature female and male *E. sinensis* (g/100 g).

Amino Acid	PFH ^1^	NFH ^1^	PFG ^1^	NFG ^1^	PMH ^1^	NMH ^1^	PMG ^1^	NMG ^1^
umami amino acid, UAA								
Asp	0.052	0.021	0.019	0.025	0.058	0.031	0.138	0.116
Glu	0.131	0.126	0.177	0.170	0.189	0.099	0.295	0.347
sweetness amino acid, SAA								
Thr *	0.088	0.051	0.109	0.124	0.102	0.053	0.051	0.040
Ser	0.016	0.004	0.001	0.001	0.018	0.009	0.012	0.031
Gly	0.128	0.077	0.129	0.078	0.145	0.103	0.215	0.137
Ala	0.230	0.102	0.154	0.078	0.271	0.149	0.303	0.211
Pro	0.203	0.134	0.259	0.256	0.234	0.151	0.414	0.591
bitterness amino acid, BAA								
Val *	0.084	0.021	0.031	0.013	0.103	0.046	0.011	0.028
Met *	0.045	0.014	0.020	0.010	0.055	0.025	0.022	0.017
Ile *	0.062	0.015	0.016	0.007	0.077	0.035	0.030	0.020
Leu *	0.125	0.037	0.028	0.012	0.157	0.080	0.034	0.028
Tyr	0.002	0.028	0.004	0.016	0.092	0.051	0.044	0.051
Phe *	0.071	0.022	0.031	0.015	0.090	0.047	0.031	0.033
Lys *	0.150	0.058	0.126	0.128	0.172	0.100	0.047	0.046
His **	0.038	0.014	0.050	0.039	0.048	0.025	0.032	0.025
Arg **	0.374	0.301	0.398	0.363	0.409	0.343	0.242	0.201
∑UAAs	0.183	0.147	0.196	0.195	0.247	0.13	0.433	0.463
∑SAAs	0.665	0.368	0.652	0.537	0.770	0.465	0.995	1.010
∑BAAs	0.951	0.51	0.704	0.603	1.203	0.752	0.493	0.449
flavor amino acid (UAAs + SAAs)	0.848	0.515	0.848	0.732	1.017	0.595	1.428	1.473
Asn	0.044	0.012	0.019	0.007	0.046	0.026	0.020	0.017
Gln	0.052	0.018	0.058	0.037	0.067	0.030	0.075	0.088
Trp	0.046	0.056	0.033	0.028	0.053	0.040	0.054	0.035
Cys-s	0.012	0.001	0.000	0.001	0.015	0.002	0.000	0.007
∑AAs ***	1.951	1.111	1.661	1.411	2.398	1.445	2.069	2.070

^1^ PFH: hepatopancreas of precocious female *E. sinensis*; NFH: hepatopancreas of normally mature female crab; PFG: gonads of precocious female crab; NFG: gonads of normally mature female crab; PMH: hepatopancreas of precocious male crab; NMH: hepatopancreas of normally mature male crab; PMG: gonads of precocious male crab; NMG: gonads of normally mature male crab. The same below. * essential amino acid, ** semi-essential amino acid, *** total amino acid (AA).

**Table 2:** The fatty acid contents of the hepatopancreas and gonads in the precocious and normally mature female and male *E. sinensis* (%).

Fatty Acid	PFH	NFH	PFG	NFG	PMH	NMH	PMG	NMG
C14:0	0.979	1.328	0.498		0.892	1.417	0.671	0.928
C15:0	0.260	0.443	0.214	0.247	0.330	0.490	0.288	0.407
C16:0	17.425	21.543	13.071	14.719	18.738	21.146	14.467	16.873
C17:0	0.199	0.301	0.271	0.285	0.334	0.301	0.521	0.387
C18:0	2.120	2.953	3.129	4.079	2.541	2.582	4.903	4.443
C20:0	0.149	0.157	0.128	0.139	0.225	0.144	0.186	0.176
C22:0	0.134	0.111	0.051	0.048	0.267	0.113	0.277	0.271
C14:1	0.264	0.240	0.073	0.075	0.163	0.262	0.119	0.148
C16:1	11.965	13.855	12.595	15.367	10.027	14.119	6.797	9.379
C17:1	0.419	0.496		0.110	0.606	0.554	0.043	0.032
C18:1n9t		0.314	0.473	0.492		0.372	0.600	0.691
C18:1n9c	39.067	32.638	34.465	28.784	36.033	30.397	27.171	25.671
C20:1	1.659	1.403	1.087	0.425	1.616	1.352	1.525	0.849
C22:1n9	1.089	0.039	0.291	0.037	1.128	0.052	0.577	0.087
C24:1	0.073	0.045	0.122	0.176	0.086	0.044	2.725	2.564
C18:2n6c	16.832	16.846	16.492	17.859	16.379	17.941	11.550	14.360
C20:2	0.920	1.096	0.831	0.842	1.332	1.130	2.147	1.639
C18:3n6	0.027	0.078	0.032	0.109	0.037	0.078	0.029	0.051
C18:3n3	2.776	1.834	3.611	2.210	2.748	1.899	2.090	1.675
C20:3n6	0.099	0.228	0.162	0.239	0.152	0.211	0.200	0.238
C20:3n3	0.233	0.255	0.241	0.246	0.293	0.257	0.594	0.613
C20:4n6	0.546	1.240	2.134	3.860	1.286	1.459	7.598	7.859
C20:5	1.299	1.149	5.286	5.938	2.378	1.812	9.591	6.776
C22:6n3	1.200	1.092	4.586	3.486	2.172	1.548	5.170	3.640
saturated fatty acid SFAs	21.27	26.83	17.36	19.52	23.33	26.19	21.31	23.49
unsaturated fatty acid UFAs	78.47	72.85	82.48	80.26	76.44	73.49	78.53	76.27
monounsaturated fatty acid MUFAs	54.54	49.03	49.11	45.47	49.66	47.15	39.56	39.42
polyunsaturated fatty acid PUFAs	23.93	23.82	33.38	34.79	26.78	26.34	38.97	36.85
∑n-3	4.21	3.18	8.44	5.94	5.21	3.70	7.85	5.93
∑n-6	17.50	18.39	18.82	22.07	17.85	19.69	19.38	22.51
PUFA/SFA	1.13	0.89	1.92	1.78	1.15	1.01	1.83	1.57
n-3/n-6	0.24	0.17	0.45	0.27	0.29	0.19	0.41	0.26

**Table 3:** The flavorful nucleotide contents of the hepatopancreas and gonads in the precocious and normally mature female and male *E. sinensis* (mg/kg).

Fatty Acid	PFH	NFH	PFG	NFG	PMH	NMH	PMG	NMG
cytidine monophosphate CMP	196	52.2	11,300	7595	288	74.3	224	27.1
adenosine monophosphate AMP	4.99	50.3	20,166	15,179	3.71	14.3	15.0	2.01
uridine monophosphate UMP	127	7.20	4354	2950	112	58.6	116	133
guanosine monophosphate GMP	3426	1433	4284	3510	1705	1330	277	120
inosine monophosphate IMP	16.6	19.6	16,026	18,637	15.0	7.73	85.7	127

## Data Availability

All the datas of transcriptome are available at NCBI SRA database (PRJNA897950). All the datas of metabolome in this study has been submitted to MetaboLights (MTBLS3686).

## Supplementary Materials

The following supporting information can be downloaded at: https://www.mdpi.com/article/10.3390/ijms241311171/s1.
