# Supplementary material for: Integrated Analysis of Transcriptomics and Metabolomics Unveil the Novel Insight of One-Year-Old Precocious Mechanism in the Chinese Mitten Crab, Eriocheir sinensis
Source: Int J Mol Sci. 2023 Jul 6;24(13):11171. doi: 10.3390/ijms241311171 (PMC10342783; doi:10.3390/ijms241311171)
Supplement: Supplementary file 1 [file ijms-24-11171-s001.zip › figure S1.pdf]

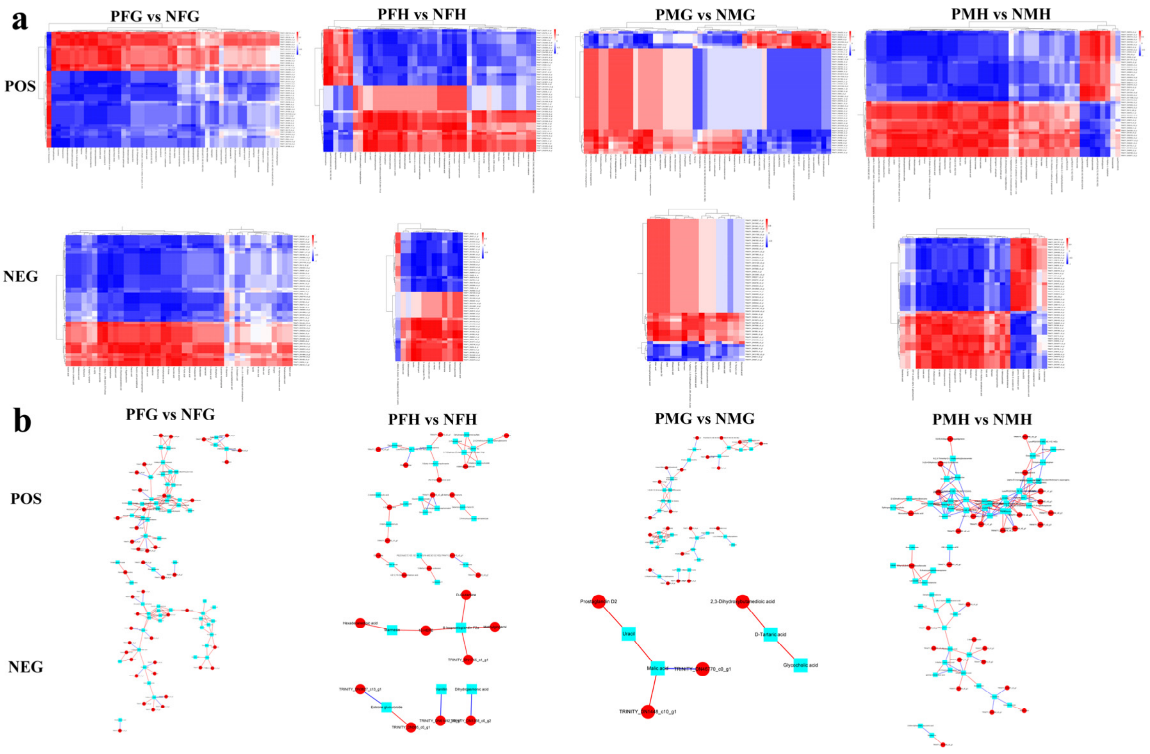

Figure S1. The spearman correlation heatmaps (a) and network regulation figures (b) of each comparison group with POS mode and NEG mode. a: The columns represent differential genes, and the rows represent differential metabolites. The magnitude of correlation is shown by different colors, where $P < 0.05$ is marked with *. b: circles are differential genes, rectangles are differential metabolites, blue lines indicate negative correlations and red lines indicate positive correlations.
